# The National Pension Fund for Nurses and Hospital Officials

**Published:** 1887-12-17

**Authors:** 


### THE NATIONAL PENSION FUND FOR NURSES AND HOSPITAL OFFICIALS.

A conference of the officials, matrons, and nurses of hospitals, infirmaries, and nursing institutions from all parts of the country, under the auspices of the Hospitals Association, was held at the Lecture Hall of the Society of Arts, John Street, Adelphi, on Thursday afternoon. Lieut.-General R. H. Keatinge, V.C. (vice-chairman of the Council), presided in the absence of Sir Andrew Clark, Bart., M.D., F.R.S., who telegraphed an apology for absence, and expressed warm sympathy with the objects of the meeting. Amongst those present were the Countess of Strafford (president of the Women's Jubilee Offering), Mrs. Burdett, and representatives of 168 institutions, from which 1,480 individual applications to join the fund had been received. Communications and representatives came from Aberdeen, Edinburgh, Glasgow, Newcastle, Hull, Leeds, Norwich, Chester, Winchester, Bristol, Plymouth, Boston, and several towns in the home counties and elsewhere, as well as from various places in Ireland and Scotland. Lady Sarah Spencer was unavoidably prevented from being present as she intended. The Duchess of Beaufort wrote from Badminton expressing regret that "she could not be in London to attend the conference, as it would have given her great pleasure to do so, and so to aid in the establishment of a Nurses' Pension Fund, a most interesting subject."

Mr. George King, the actuary of the Atlas Assurance Company, who had prepared the necessary tables, explained that all who joined the sick fund must join the annuity fund also, but anyone might be an annuitant without entering for sick pay. He had no hesitation in saying that the pension fund, on the basis of the tables as explained to the conference in detail, might be safely commenced without any other help than its own financial strength. At the time same it was highly desirable that a bonus fund should be formed of contributions from those who took an interest in the welfare of workers amongst the sick, so that an increased provision might be given to the members who showed a desire to help themselves. The fund, as elaborated with Mr. Burdett's assistance, was now organised upon a perfectly sound basis, and everyone might join it with perfect confidence.

Mr. Burdett said, amidst cheers, that they owed a debt of gratitude to the Countess of Strafford for her attendance that day to show her great sympathy with those engaged in nursing the sick. Lady Strafford had indeed shown a deep interest in the pension fund scheme. Sir Andrew Clark had applied to the Queen to set apart £20,000 of the Jubilee offering to enable them to commence the fund on a more adequate basis than the Friendly Society's Acts allowed. The fund should be constituted under the Life Assurance Act of 1870, and that was why application was made for the £20,000. Sir Andrew had been referred to the committee appointed to prepare a scheme for the disposal of the £75,000 surplus of the Jubilee offering. It was feared, however, that the application would be found to have been made too late, as the committee had been constituted some time before the application reached them. A decision had been come to, but its nature was not yet publicly known. It was satisfactory to hear from the actuary how perfectly sound the fund would be, whether outside help came or not. He believed, in any case, that four or five, or possibly even one lady or gentleman of wealth would be found willing to place £20,000, or £4,000 or £5,000 each, as the case might be, in the hands of the Accountant-General in Chancery, in compliance with Section 3 of the Act, to be invested in their own names as trustees for the National Pension Fund until the premiums amounted to £40,000, when the deposit could be withdrawn. The deposit of £20,000 would be locked up, at the outside, for ten years, as the actuary would tell them, and meantime the depositor or depositors could receive the dividends at their option. Mr. King had shown that a special pension fund was necessary if the nurses were to be adequately provided for, because no existing agency could meet the special requirements and circumstances of the workers among the sick, whose earnings were relatively so small as to need the encouragement and sympathy as well as the support of their employers and the public at large.

An interesting discussion followed, in which Colonel Brooshooft, Surgeon-Major Ince, M.D., and several of the officers present took part.

In the result it was determined to request the Council of the Hospitals Association to make the necessary arrangements for commencing the fund at once. Some amusement was caused by the fact that several of the representatives from country institutions had brought their money with them to pay into the fund there and then.

A vote of thanks to the chairman terminated an interesting and successful conference.—*Times.*

